# Extra-Limites

**Published:** 1837-04-01

**Authors:** 


### EXTRA-LIMITES.

### Irish Medical Profession.

At a general meeting of the physicians and surgeons of the province of Munster, lately held in Cork, a very decided and judicious step was taken, and one that, if we mistake not, is likely to be productive of very beneficial consequences—we allude to the appointing of a deputation, consisting of Dr. Nugent of Cork, and Surgeon Phelan of Clonmel, (author of a late work on "the Defects and Abuses of the Medical Charities of Ireland, with Suggestions for a Medical Poor-law,") which was instructed to attend in London, whilst the Irish Poor-law Bill, or any other likely to affect the interests or respectability of their body, should be under the consideration of Parliament. These gentlemen have been some time in London, most actively engaged in pointing out for the consideration of members of Parliament, the urgent necessity there is for making a change in the laws relating to the public medical institutions of Ireland, and the most advisable mode of doing so.

That this subject is a very important one, and that immediate legislation on it is imperatively called for, the following, which will be found a correct statistic on Irish public charities, clearly prove. There are in Ireland about 618 medical charities, supported partly, or entirely, by public funds. They cost, at present, (taking the average of ordinary years, when there are no serious epidemics,) between £170,000. & £180,000. per annum. This fund is supplied by Parliamentary and Treasury grants of £20,000., by county presentments of nearly £90,000., produce of property accumulated, and fines at petty sessions, £25,000.; subscriptions and donations, £40,000.; total, £175,000. a year.

The number of sick, to whom medical aid is annually afforded, by means of these charities, is above one million, viz. interns in infirmaries and from hospitals, 20,000 in the former, and 16,000 in the latter; externs, attended to by dispensaries and infirmaries, *about* 968,000 by the former, and 130,000 by the latter; total, 1,134,000.

We had the pleasure of conversing with Messrs. Nugent and Phelan, and find these to be the views of those who sent them, as well as their own.

They recommend the appointment of a Commission, composed of two medical men, and one non-professional person, (if the Government or Parliament wish it so,) with two or three medical inspectors, to whom the entire management of this great and important mass of institutions is to be solely entrusted. They state, and we think very truly, that such a department should only be in the hands of persons capable of understanding its numerous difficulties and details; and that there are many reasons, in which we also agree, why, in Ireland especially, the relief for the destitute poor, and that for the sick, should not be in the same hands.

We feel very great satisfaction in learning that Mr. Nicholls, who at first was disposed to view the matter differently, now completely concurs with the deputation, and that, after some interviews were had between them, that gentleman, much to his credit, has strongly expressed his accordance with the opinions of Messrs. Nugent and Phelan, who are still in communication with Lord Morpeth and Mr. Nicholls on the subject. We shall only add that, were the medical profession in other parts of the empire to act in the same manner, they would soon find what advantages would result from it. We therefore strongly recommend a similar line of policy to the other provinces in Ireland, as well as to our nearer neighbours, who may well take an example from the profession in that country.

We understand the deputation has given Lord Morpeth the heads of a Bill, but it cannot be made public at present.

### THE EFFICACY OF LOBELIA INFLATA IN INFLAMMATIONS AND CONGESTIONS OF THE MUCOUS LINING OF THE BRONCHIAL TUBES.

*To Henry James Johnson, Esq. Lecturer on Anatomy, in the School of Kinnerton Street.*

Dear Sir,—I perceive from the Journals that a disease called Epidemic Catarrh or Influenza, is very fatal in London—that it is attended with inflammation of the mucous membrane of the bronchial tubes—bears depletion badly—and that the old and infirm are the greatest sufferers. The experience and observation of a number of years devoted to the practice of medicine, have led me to two important conclusions in regard to the principal diseases of the chest.

1st. That when the serous membranes of the chest are affected, tartar emetic and the lancet are worth all other remedies put together, and those cases which will not bear the lancet, will bear tartar emetic in considerable doses.

2nd. That in those diseases affecting the mucous lining of the bronchial tubes, the lobelia inflata comes as near being a specific as tartar emetic, and the lancet in pneumonia and pleurisy. As a remedy for asthma you are of course acquainted with the virtues of the lobelia inflata. The observation, that it holds a controlling power over inflammations of the mucous membrane of the lungs, has never, I believe, been announced to the medical world, and originated with myself. I always intended to make it known to the public. My notes of the efficacy of this remedy I left in the United States. If I had them, I could give you more definite and precise information in regard to the therapeutic properties of the lobelia in such affections. But I have thought that a bare enumeration of the principle, that lobelia inflata is almost a specific for inflammations and engorgements of the mucous membrane of the bronchi, and for spasmodic constriction of those tubes, might be attended with considerable benefit at the present crisis. I have very little doubt, that if the remedy be tested in your present epidemic, experience will establish the principle beyond cavil or dispute,—*that lobelia inflata is, for inflammations and congestions of the mucous coat of the bronchial tubes, precisely what the lancet and antimonials are for inflammations of the serous membranes of the thoracic viscera.* I have never found venesection, antimonials, or purgatives of much utility in severe catarrhal fevers. Used with moderation, the effects of such remedies are often equivocal, and in excess, always prejudicial. You have, no doubt, observed, that in violent catarrhal fevers, the mucous lining of the alimentary canal sympathizes with the inflammation of the mucous membrane of the lungs. Hurried respiration, red tongue, weak and frequent pulse occuring in catarrhal affections, especially in debilitated children and in old persons, always indicate considerable danger. This is precisely the pathological state of the system, which the lobelia inflata is so well calculated to remove. Its action is so prompt and self-evident, that there can be no mistake about its virtues. It is altogether a different medicine in this state of the system, from what it is when given to a person in health or differently affected. In the healthy state the lobelia is powerfully emetic, but in the inflamed, conjested, or spasmodic state of the bronchial tubes, it neither vomits, nor produces any sensible effect upon the great organs of secretion. It seems to spend its force in diminishing the anhelation, in lessening the frequency of the pulse, in allaying the general febrile commotion, and in restoring the balance of the circulation and excitability. Why it should do so, is as difficult to explain, as why opium is not a narcotic when it has pain or spasm to allay. I use a saturated tincture made of the leaves, stems, and inflated capsules of the plant. Dose—one or two teaspoonsful every two or three hours, oftener, or not so often, according to the urgency of the symptoms. I mix it with about an equal quantity of the syrup of squills, or the oxymel. I have given to children, less than a year old, from 12 to 20 drops, without producing vomiting. When the remedy vomits, it indicates that the dose is either too large, or the condition of the system is not applicable to its employment. It produces its best effects when it creates no visible constitutional disturbance. When the anhelation comes on in paroxysms, a dose or two of quinine may be safely and advantageously used in the intermission, and the lobelia in the paroxysms.

Most respectfully, your
Obedient Servant,
SAMUEL A. CARTWRIGHT, M.D.
Of Natchez, Mississippi.

---

### SELECTIONS FROM THE LAWS AND REGULATIONS OF THE BRITISH MEDICAL ASSOCIATION.

### OF THE QUALIFICATIONS AND ADMISSION OF MEMBERS.

1.—EVERY legally qualified practitioner in medicine may become a member, on producing to the Secretary satisfactory evidence of qualification.

2.—Every member on his admission after payment of his first contribution, shall be presented with a copy of the laws, and shall sign the following declaration, in a book kept for this purpose, viz.:—

"We, whose names are hereunto subscribed, promise that we will obey the laws and regulations of the British Medical Association, so long as we shall continue members thereof."

3.—If the member admitted should not be able to attend in person, he shall, by letter, authorise the Secretary to sign the declaration on his behalf; and such letter shall be preserved among the papers of the Association.

4.—No person practising any secret mode of treatment, and no proprietor or advertiser of any empirical nostrum, shall become a member of the Association.

### OF CONTRIBUTIONS, &c.

1.—Each member shall subscribe annually the sum of £1. 1*s*. Subscriptions to become due on the 1st day of January in each year.

2.—Each member shall, on admission, pay a proportion of the annual subscription,—to be computed from the quarter day nearest such admission.

### OF THE OFFICERS AND COUNCIL.

1.—To carry into effect the various important objects of the Association, and to manage its affairs, there shall be elected annually, in September, a Treasurer, three Auditors, and thirty-two Councillors. These, with the chairman of every County Branch Association (who shall, *ex officio*, be a member, if a General Practitioner) shall constitute the Council.

2.—At the first meeting of the Council, after its election, its members shall elect from among themselves, a President, six Vice-Presidents, and a Secretary. All the officers and Councillors shall be, or shall have been, General Practitioners.

### SECT. I.—*Of the President and Vice-Presidents.*

1.—The President shall always be a General Practitioner; he shall take the chair at all meetings, when present; and shall regulate the proceedings, preserve order, and enforce the laws of the Association.

2.—He shall state and put all questions at such meetings; and shall have a casting vote only.

3.—He shall also direct the summoning of all extraordinary meetings of the Association or Council; and shall sign the minutes of all meetings, after they have been confirmed.

4.—In the absence of the President, the chair shall be taken by the Vice-President, then present, whose name stands highest in the lists. If all should be absent, then by the Treasurer, by one of the Auditors, or by the senior Councillor present.

### SECT. II.—*Of the Treasurer and Auditors the duties are the usual ones.*

### SECT. IV.—*Of the Council and its Meetings.*

1.—The Council shall meet on the second Tuesday in every month (excepting in September, and then on the first Tuesday) at 7 o'clock in the evening precisely; and shall have the power of adjournment to any future day or hour. Six members to be a quorum for the transaction of business.

2.—An extraordinary meeting of the Council may at any time be held, by order of the President, provided the Secretary shall have given notice of the time and object of such meeting to all its members residing within the Metropolitan district.

3.—The Council shall have the power of regulating, until the next general meeting, the duties of any of the Officers, when these are not accurately defined by the laws.

4.—The Council shall regulate all the expenditure of the Association.

5.—The Council shall have a discretionary power to take such steps for the suppression of quackery, and of the illegal and dangerous practice of medicine and surgery by unqualified persons, as the law authorizes, and as may seem to be expedient.

6.—The Council shall also possess a discretionary power to adopt such measures in accordance with the general laws, as it may at any time deem necessary for the preservation and extension of the rights, privileges, and immunities of the Association, and of its members; and for the support of the honour, welfare, and respectability of the medical profession.

7.—The Council shall be empowered to make such bye-laws for the governance of its own body, and of Committees, as it shall from time to time think necessary.

8.—If any member shall be aggrieved by the practice of a person not legally qualified; or by the institution of any unjust prosecution, action, or other legal process against him, he shall send his complaint, with the best evidence he can procure, to the Secretary, who shall submit it to the next meeting of the Council.

9.—The Council shall be empowered to employ legal assistance for the protection of any member of the Association, who may improperly or unjustly be made the subject of prosecution, action, or other legal process; or to adopt such other measures as it may consider expedient.

10.—All questions shall be decided in the Council by a show of hands, or division; and accurate lists of all divisions shall be made, and entered in the minutes.

11.—The proceedings of the Council shall be recorded in a book kept for the purpose by the Secretary; which shall be open to the inspection of the members of the Association, under such regulations as the Council may determine.

### SECT. V.—*Of the Secretary nothing unusual.*

### OF THE ELECTION OF THE COUNCIL.

1.—Any two members may send to the Secretary, before the ordinary meeting in September, the names of any members residing within the Metropolitan district, whom they may propose as Officers and Councillors, instead of those who are to retire; provided they shall first have obtained their consent to serve, if elected.

2.—These names shall be submitted by the Secretary to the next ordinary meeting; and those of persons eligible shall be published in the medical, and such other, periodicals, as the council may appoint, at least fourteen days before the election.

3.—Every member may vote for any of the names in the published list, and deliver or send them to the Secretary, free of expense, on or before the 27th day of September, in a sealed paper; which, if sent, shall be enclosed in another, having the voter's name written within it.

4.—The President may be re-elected; but shall at no time be permitted to preside more than two years in succession.

### OF GENERAL MEETINGS OF THE ASSOCIATION, AND OF ALTERATIONS OF THE LAWS.

1.—There shall be two general meetings in the metropolis, every year; the first on the Monday preceding the 25th day of March, and the second (the anniversary) on the 30th day of September.

2.—The first of these meetings shall be held for making such alterations of, and additions to the laws, as may be considered necessary according to clause 6th of this Chapter, and for the transaction of any business that may be brought before it.

3.—At the second, the result of the ballot for the Council shall be declared; a general report of the state of the Association shall be presented from the Council; and a financial one from the Auditors. Any other business (except alterations of the laws) may be transacted.

### OF ORDINARY MEETINGS.

1.—After every monthly meeting of the Council, an ordinary meeting of the Association shall be held at 9 o'clock P.M. for the admission of new members, and such other business as the Council may direct.

### OF BRANCH ASSOCIATIONS AND DISTRICT COMMITTEES.

1.—The Council shall endeavour to promote the formation of Branch Associations, and District Committees, in every county, city, town, and district of sufficient importance, throughout the Kingdom, beyond the limits of the Metropolis.

2.—Each member of a Branch Association shall be considered a member of the British Medical Association, and shall pay annually, on the first day of January, the sum of £1. 1*s*. to the Treasurer of his branch; one half of which shall be transmitted to the general fund of the Association; the other half shall be retained to defray the necessary expences of the Branch Association: the surplus, if any, to be appropriated annually to the general fund.

3.—No Branch Association or District Committee shall commence any prosecution, or enter upon any proceeding of importance to the general body, without having first obtained the sanction of the Metropolitan Council, or of a general meeting of the Association.

### Of the Benevolent Fund.

1.—A Benevolent Fund shall be established, for the purpose of affording relief to decayed members of the Medical profession, and to their widows and orphans.

2.—Three trustees shall be appointed by a general meeting, to hold the Benevolent Fund in trust for the purpose intended; and all investments shall be made, all securities taken, and all stock purchased in the name of the Trustees for the time being, according to the discretion and under the control of the Council, which shall have the management and disposition of the subject matter of the trusts.

3.—If any Trustee shall become bankrupt or insolvent, or from any other cause become incapable of well and duly performing the duties of the trust, he shall cease to be such Trustee accordingly; and if the Council at any time see cause to remove any Trustee, on the ground that he does not well and truly perform the duties of the said trust, he shall, upon a declaration of the Council to such effect, cease to be a Trustee; and when, from any of the aforesaid causes, or by death or otherwise, the number of Trustees shall be less than three, the Council shall fill up the vacancy, subject to the approbation of the next half-yearly general meeting.

4.—Every order of the Council signed by the Chairman and Treasurer, and countersigned by the Secretary, shall be a sufficient warrant to the Trustees for the time being, for any investment, purchase, sale, or disposition of the stock or monies subscribed for the formation and support of the Benevolent Fund.

5.—No relief shall be afforded to any person from the Benevolent Fund, until it shall amount to £2000., when regulations as to the mode of distributing the interest arising from it, shall be made at a general meeting to be held for that purpose.

---

*Remarks.*—Possibly the object of the following few remarks may have ceased to exist before this number of the Journal appears. It is evident that the Association, as it now stands, is designed to be one of General Practitioners only, and should have been so designated from the beginning. But a most impolitic, perhaps fatal step has been taken. The Association admits all legally qualified practitioners as *members;* and afterwards prohibits all physicians and surgeons not practising or having practised pharmacy, from becoming *officers* of the institution, or taking any part in its *councils!!* Yet this is the Association which declaims against an analogous course pursued by the College of Surgeons! The step may be retraced, but the mischief is nearly irretrievable. The insult having been offered to physicians and surgeons, they of course, will take no interest in the Institution. The only wise plan open to the Association is to call themselves "The British Association of General Practitioners"—and only to admit as members the said General Practitioners. By this procedure they give no offence to any class, and they will be acting openly, honestly, and straight-forward. Let them take the "Law Association" as an example. That association admits only attorneys and solicitors. They do not invite barristers to become members, and prohibit them from taking part in their councils. They do not receive funds from *members*, who are denied a voice in the distribution of them as councillors.

---

### Case of Iritis and Temporary Blindness, produced by Tic Douloureux. By D. Jamison, M.D.

[*Communicated by Dr. Johnson.*]

W—— D——, a labourer, aged 34, never had rheumatism, gout, or syphilis; and never took mercury, until his eye became diseased. After being subject, for some time, to tic douloureux on the left side of the forehead and face, the eye of that side became inflamed. He was afterwards seized with severe pain in the eye, intolerance of light, and almost total loss of vision. In this state he applied for medical advice. He took mercury—was leeched, blistered, &c. without benefit. When the disease had existed about two months, he came under my care. At that time, the conjunctiva was inflamed ; there were two or three opaque spots on the cornea,—the iris was changed in color—the pupil had not the shining blackness of the other—its margin was indistinct—and it was immoveable. The sclerotic was not inflamed ;—he could do little more than distinguish night from day, with the affected eye—while I was examining him, he had a paroxysm of tic douloureux. The orbicularis palpebrarum contracted spasmodically—tears flowed down the cheek—and the conjunctiva became much redder. I gave him a dose of sulphate of magnesia ; then began the use of carb. ferri, in large doses. He took a small quantity of the sulph. mag. every morning, and bathed the eye frequently with an astringent lotion. In a short time, the attacks of tic douloureux became much less frequent, and much less severe. They then departed, and with them the inflammation of the conjunctiva; but the sight was not improved. I now gave him the tincture of iodine. After some time, the iris regained its color—the pupil became dilatable—the specks on the cornea disappeared, and vision was perfectly restored.

---

### RECLAMATIONS.

Every author possesses the undoubted right to protest against the observations of Reviewers. The critic must submit to be criticised, and if he executes his office in a partial, an ignorant, or an overbearing manner, he merits criticism's most indignant lash.

We cannot be accused of refusing to any author ample opportunity for dissenting from our expressed opinions on him. We seldom reply to any reclamation, being content to allow the public to decide, and willing to accord to the aggrieved party the benefit (no slight one) of the last word.

But latterly it has been the fashion for a certain class of people, when their niaiseries are exposed, to turn round on the Reviewer, abuse him like pickpockets, and vent their rage, disappointment, and vulgar vituperation, in one or more of the weekly Journals. This is so contemptible that it would not deserve notice, were it not for the truculent spirit that it fosters, and for its evil consequences on the bearing of professional men.

We have have been led to make this brief observation in consequence of some letters directed against us, which have made their appearance in the Lancet. The writers have conveniently shrouded themselves under fictitious designations, and "Castigator," an "Old Practitioner," &c. have honoured us by their abuse. The execution of these letters is as miserable, as the motives of the writers are malicious. We are denounced as enemies to the General Practitioners. To this we condescend to make but one reply. Our aim is to benefit them and all other classes of our professional brethren, by the dissemination of scientific knowledge, of liberal but not extravagant political principles, and, last not least, of gentlemanly feeling. Such is the *spirit* in which we write, and that spirit will not be judged by insulated expressions nor by garbled sentences.

Another word and we have done. Mr. Wallace, of Dublin, has also honoured us by *his* abuse. Brandishing before a wretched class, a Number of the Medico-Chirurgical Review, he has affected to see in it the signs of dissolution. We shall not throw away our labour by recommending Mr. Wallace to imitate the conduct of a gentleman. But we are quite sure that, to play the mountebank is neither the way to support an argument, nor to rise in the estimation of men of sense.

